# PPAR-*γ* Activation Prevents Septic Cardiac Dysfunction via Inhibition of Apoptosis and Necroptosis

**DOI:** 10.1155/2017/8326749

**Published:** 2017-08-03

**Authors:** Shiyan Peng, Junmei Xu, Wei Ruan, Suobei Li, Feng Xiao

**Affiliations:** Department of Anesthesiology, The Second Xiangya Hospital, Central South University, Changsha, Hunan 410011, China

## Abstract

Sepsis-induced cardiac dysfunction remains one of the major causes of death in intensive care units. Overwhelmed inflammatory response and unrestrained cell death play critical roles in sepsis-induced cardiac dysfunction. Peroxisome proliferator-activated receptor- (PPAR-) *γ* has been proven to be cardioprotective in sepsis. However, the mechanism of PPAR-*γ*-mediated cardioprotection and its relationship with inflammation and cell death are unclear. We hypothesized that activation of PPAR-*γ* by reducing cardiac inflammation, myocardial apoptosis, and necroptosis may prevent myocardial dysfunction in sepsis. Rats were subjected to cecal ligation and puncture (CLP) with or without PPAR-*γ* agonist (rosiglitazone) or antagonist T0070907 (T007). After CLP, cardiac function was significantly depressed, which was associated with the destructed myocardium, upregulated proinflammatory cytokines, and increased apoptosis, necrosis, and necroptosis. This process is corresponded with decreased inhibitor *κ*B (I*κ*B*α*) and increased NF-*κ*B, receptor-interacting protein kinase-1 (RIP1), RIP3, and mixed lineage kinase-like (MLKL) protein. Activation of PPAR-*γ* by rosiglitazone pretreatment enhanced PPAR-*γ* activity and prevented these changes, thereby improving the survival of septic rats. In contrast, inhibition of PPAR-*γ* by T007 further exacerbated the condition, dropping the survival rate to nearly 0%. In conclusion, PPAR-*γ* activation by reducing proinflammatory cytokines, apoptosis, and necroptosis in the myocardium prevents septic myocardial dysfunction.

## 1. Introduction

Sepsis is recognized as a systemic dysregulated inflammatory response to infection or injury causing life-threatening multiple organ dysfunction [1, 2]. Cardiac dysfunction is present in more than 50% of septic patients and is the major contributor to the increase of mortality in subjects with sepsis [3, 4]. Despite rigorous efforts from researches worldwide pointing out the important role of inflammation in sepsis, its specific role in the pathophysiology of septic cardiac dysfunction have not been fully elucidated and current effective treatment is lacking [5].

Septic cardiac dysfunction is frequently associated with imbalance in production of proinflammatory cytokines [6, 7], including tumor necrosis factor-*α* (TNF-*α*) and interleukin 6 (IL-6), which leads to myocyte death and causes microlesions in the myocardium, hence myocardial dysfunction [8, 9]. Studies have demonstrated that reducing inflammatory cytokine production attenuates cardiac dysfunction in sepsis [10], which highlights the critical role of inflammation in the treatment of cardiac dysfunction in sepsis. In recent decades, peroxisome proliferator-activated receptor gamma (PPAR-*γ*), a ligand-activated transcription factor that is involved in cell proliferation, lipid metabolism, and inflammation [11], emerges as a regulator of inflammatory responses [12]. Increase or activation of PPAR-*γ* has been shown to improve survival in animal models of sepsis [13, 14]. In the cecal ligation and puncture (CLP) model of sepsis, PPAR-*γ* agonist ameliorates systemic inflammation by decreasing plasma levels of TNF-*α* and IL-6 via inhibition of nuclear factor kappa B (NF-*κ*B) [15, 16]. However, in the lipopolysaccharide (LPS) model of sepsis, while overexpression of PPAR-*γ* in cardiomyocyte prevents septic cardiac dysfunction, cardiac mRNA levels of interleukin 1*β* (IL-1*β*), IL-6, and TNF-*α* are not reduced [17]. It was suggested that PPAR-*γ*-mediated improvement of cardiac function is not associated with alleviation of local cardiac inflammation at the early stage of sepsis [17, 18]. Nonetheless, all these support the notion that PPAR-*γ* activation is cardioprotective against sepsis-induced cardiac dysfunction. However, the underlying mechanism, especially the role of inflammation in sepsis-induced cardiac dysfunction, is still unclear.

As adult cardiomyocytes are nonproliferative, the loss of cardiomyocytes due to apoptosis or necrosis have been considered as the underlying mechanism in the development of cardiac dysfunction after sepsis [10, 19, 20]. Interestingly, necroptosis, a newly identified programmed cell death, which comprises characters of apoptosis and necrosis, is considered a proinflammatory form of cell death [21]. Given the importance of proinflammation activation in the development of cardiac dysfunction in sepsis and that necroptosis can activate apoptosis [22], it is possible that necroptosis may play a role in sepsis-induced cardiac dysfunction. We, therefore, hypothesized that activation of PPAR-*γ* may reduce inflammation, thereby inhibiting myocardial apoptosis and necroptosis and ameliorating cardiac dysfunction during sepsis. To test this hypothesis, rats with CLP-induced sepsis were treated with PPAR-*γ* agonist or antagonist, and the proinflammatory and apoptotic/necrotic parameters in the myocardium were assessed.

## 2. Materials and Methods

### 2.1. Animals

Male pathogen-free Sprague Dawley rats, weighing between 200 and 220 g, were used for the current study. All the rats were kept under optimum conditions (24 ± 1°C, 50 ± 10% humidity, and 12 h/12 h dark-light cycle) at the animal unit of the Central South University laboratory and were fed ad libitum with standard pellet diet and water. The procedures were reviewed and approved by the Central South University Local Committee on Animal Research Ethics. The animal experiments were performed according to the guidelines for the care and use of animals established by Central South University.

### 2.2. Experiment Design

Twenty-four rats were randomized into the following four groups: the sham group (*n* = 6), the CLP group (*n* = 6), the PPAR-*γ* agonist rosiglitazone-treated group (CLP + ROT group, *n* = 6), and the PPAR-*γ*-selective inhibitor T0070907 group (CLP + T007 group, *n* = 6). Rosiglitazone (10 mg/kg, i.p., Sigma-Aldrich, CA, USA) or T0070907 (1.5 mg/kg, i.p., Selleck Chemicals, Houston, TX, USA) or sterile normal saline was administered 60 minutes before surgery. A cohort of animals (*n* = 16 per group) receiving the same protocols were used to assess survival rates. The survival rate was evaluated within 72 hours in each group.

### 2.3. Cecal Ligation and Puncture (CLP) Model

After a 7-day adaptation and subsequent 12-hour deprivation of food, all rats received cecal ligation and puncture (CLP), a classic sepsis model, except those in the sham group. After anesthetized with isoflurane (2%) using an anesthetic mask, the skin of the surgery field was sterilized and a midline of 3 cm abdominal incision was made to expose cecum which was then ligated between the ileocecal valve and terminal. The cecum was punctured twice “through and through” on the antimesenteric border with a 16-gauge (1.65 mm in diameter) needle, and a small amount of cecal contents was squeezed out through the puncture wound [23]. Finally, the cecum was restored into the abdominal cavity. The incision was then sutured layer by layer with a 4–0 silk suture. Comparatively, rats in the sham group received laparotomy and intestinal canal exposure but without ligation and puncture. Each rat received normal saline in a volume of 4 mL/100 g by intraperitoneal injection immediately after CLP. A catheter filled with heparin saline was inserted into the left ventricle at 18 hours after CLP to measure the maximal positive and negative peak of first derivative of LV pressure (+dP/dt_max_, −dP/dt_max_), the left ventricle peak systolic pressure (LVSP), and the left ventricle end diastolic pressure (LVEDP) as described previously [24].

### 2.4. Pathological Assay and Serum Biochemical Test

Animals were sacrificed using carbon dioxide (CO_2_) inhalation method at 18 hours after CLP. Left ventricular myocardial tissues were harvested and stained with hematoxylin-eosin (H&E) staining as previously described [25]. Serum samples were collected from the abdominal aorta, and creatine kinase isoenzyme (CK-MB) (Shanghai Jianglai Bioengineering Institute, Co., Ltd., Shanghai, China) and lactate dehydrogenase (LDH) (Nanjing Jiancheng Bioengineering Institute, Nanjing, China) concentrations were detected according to the manufacturer's instructions.

### 2.5. PPAR-*γ* DNA-Binding Analysis

The myocardial tissue nuclear proteins were separated according to the protocol described in the NE-PER kit (Thermo Scientific, USA). Nuclear proteins PPAR-*γ* DNA-binding activities were analyzed according to PPAR-*γ* transcriptional activity using ELISA assay kit (Cayman Chemical, MI, USA).

### 2.6. TUNEL Staining

Apoptotic cells in the myocardial tissue paraffin sections were identified by the terminal deoxynucleotidyl transferase-mediated dUTP nick end labeling (TUNEL) technique, according to manufacturer's instructions (Roche Applied Sciences, Shanghai, China). TUNEL-positive myocytes were analyzed using light microscopy (Leica Microsystems Digital Imaging, Cambridge, UK).

### 2.7. Immunohistochemistry

Myocardial tissue was fixed in 10% formalin and embedded in paraffin. Paraffin-embedded tissues were then sectioned at a thickness of 5 *μ*m. Sections were deparaffinized, hydrated, and incubated with primary PPAR-*γ* antibody (Cell Signaling Technology, Danvers, USA, diluted 1 : 500) overnight, followed by incubation with horseradish peroxidase-coupled anti-rabbit IgG antibody for 2 hours and then incubation with diaminobenzidine (DAB) for 3 minutes. Phosphate buffer saline (PBS) was used to replace the primary antibody in the negative control. DAB staining intensity was observed under a light microscope (Leica Microsystems Digital Imaging, Cambridge, UK) and assessed with a microscopic image analysis system (ImageJ, National Institutes of Health, USA).

### 2.8. Real-Time PCR Analysis

RNAs were extracted from the homogenized myocardial tissue according to the manufacturer's instruction with TRIzol Reagent (Invitrogen). Using the standard protocols described previously [26], total RNA was reverse-transcribed into cDNA. Real-time PCR were performed in a LightCycler 480 (Roche, Mannheim, Germany) to probe IL-1*β*, IL-6, TNF-*α*, and GAPDH. PCR experiments were performed using the light cycler DNA master SYBR green I kit (Roche Molecular Biochemicals, Mannheim, Germany) as described previously [26]. The following primers were used: IL-1*β* sense: 5′-CACCTTCTTTTCCTTCATCTTTG-3′, IL-1*β* antisense 5′-GTCGTTGCTTGTCTCTCCTTGTA-3′; IL-6 sense 5′-TGATGGA TGCTTCCAAACTG-3′, IL-6 antisense 5′-GAGCATTGGAAGTTGGGGTA-3′; TNF-*α* sense 5′-ACTGAACTTCGGGGTGATTG-3′, TNF-*α* antisense 5′-GCTTGG GGTTTGCTACGAC-3′; GAPDH sense 5′-GTATTGGGCGCCTGGTCACC-3′, and GAPDH antisense 5′-CGCTCCTGGAAGATGGTGATGG-3′. The relative expression of the target genes was calculated using the 2^−ΔΔ^Ct method described in a previous study [26].

### 2.9. Western Blot Analysis

Myocardial tissues were homogenized and extracted and then boiled for 10 minutes to denature proteins. Nuclear proteins were then separated according to the protocol described in the NE-PER kit (Thermo Scientific, USA). Denatured protein samples were loaded on 12% sodium dodecyl sulfate polyacrylamide gel electrophoresis (SDS-PAGE) in running buffer, then transferred to a nitrocellulose membrane at 4°C for 2 hours at 200 mA in a transfer buffer. After blocking with 5% nonfat dry milk for one hour at room temperature, the nitrocellulose membrane was incubated with primary antibodies I*κ*B (Cell Signaling Technology, Danvers, USA, diluted 1 : 1000) or NF-*κ*B p65 (Cell Signaling Technology, Danvers, USA, diluted 1 : 1000) or RIP1 (Cell Signaling Technology, Danvers, USA, diluted 1 : 1000) or RIP3 (Cell Signaling Technology, Danvers, USA, diluted 1 : 1000) or MLKL (Cell Signaling Technology, Danvers, USA, diluted 1 : 1000) overnight at 4°C. Then, the membrane was incubated with secondary antibody at room temperature for 1 hour, and the target signal was visualized with the ECL exposure system. The films were scanned and analyzed using the image analysis system (ImageJ, National Institutes of Health, USA). The contents of proteins were normalized to that of beta-actin or LaminB2. All experiments were repeated in triples.

### 2.10. Statistical Analysis

Data are presented as means ± standard error of the mean (SEM) and analyzed by the statistical software package SPSS 13.0. Differences among all groups were analyzed using *one-way analysis of variance*, followed by *Tukey* post hoc *test*. *P* values *<0.05* were considered statistically significant. Animal survival rates were analyzed by the *Kaplan-Meier* test.

## 3. Results

### 3.1. Activation of PPAR-*γ* Prevents Sepsis-Induced Cardiac Dysfunction

**Figure 1:**
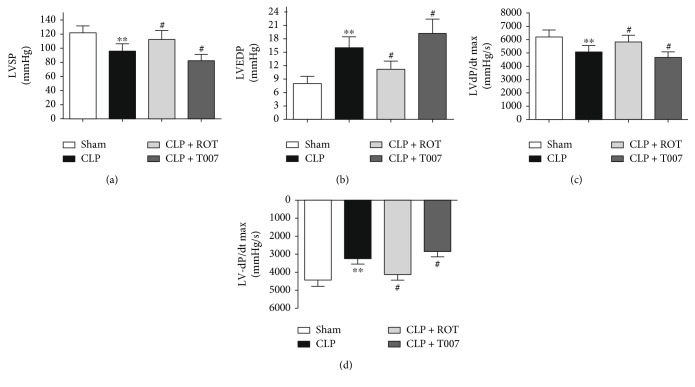
Indices of cardiac systolic and diastolic function by a pressure-volume conductance catheter system in septic rats. PPAR-*γ* agonist rosiglitazone (ROT) or antagonist T007 was injected intravenously 1 hour before CLP. Cardiac function measured 18 hours after CLP was demonstrated in terms of (a) left ventricular systolic pressure (LVSP), (b) left ventricular end diastolic pressure (LVEDP), (c) maximal slope of left ventricular systolic pressure increment (LV dP/dt_max_), and (d) maximal slope of left ventricular diastolic pressure decrement (LV −dP/dt_max_). ^∗∗^*P* < 0.01 versus sham. ^#^*P* < 0.05 versus CLP. Data are presented as mean ± SEM. *n* = 6 per group.

To assess whether activation of PPAR-*γ* prevents sepsis-induced cardiac dysfunction, PPAR-*γ* agonist rosiglitazone or antagonist T007 was injected intravenously 1 hour prior to CLP surgery. CLP-induced sepsis severely compromised cardiac function, evidenced by reduced dP/dt_max_, −dP/dt_max_, LVSP and increased LVEDP (CLP versus sham, *P* < 0.01) (Figure 1). Administration of rosiglitazone prevented cardiac dysfunction induced by CLP (CLP + ROT versus CLP, *P* < 0.05). In contrast, pretreatment with the PPAR-*γ* inhibitor, T007, aggravated septic cardiac dysfunction (CLP + T007 versus CLP, *P* < 0.05).

### 3.2. PPAR-*γ* Activation Improves Survival in Rats with Sepsis

**Figure 2:**
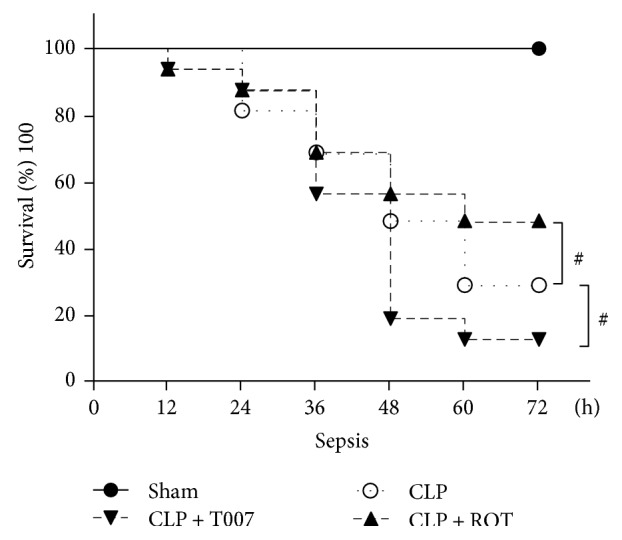
Survival rates of rats after CLP. Rats were subjected to CLP with or without pretreatment of rosiglitazone (ROT) OR T007 and were monitored for survival for 72 hours. *n* = 16 per group. ^#^*P* < 0.05 versus CLP.

To determine the role of PPAR-*γ* on animal survival after CLP, animals were treated with an intravenous injection of PPAR-*γ* agonist rosiglitazone or PPAR-*γ* inhibitor T007 before CLP and were monitored for 72 hours. We first observed a slight decrease in the survival rate 12 hours after CLP in T007 and rosiglitazone-treated animals. From 24 hours onwards, survival rates of the CLP and CLP + T007 groups dropped drastically from about 80% to 30% and to 15%, respectively, by 72 hours. In contrast, rosiglitazone increased the survival rate to around 50% by a 72-hour end point. No mortality was observed in sham-operated animals (Figure 2).

### 3.3. Activation of PPAR-*γ* Prevents Myocardial Injury and Necrosis in Septic Rats

**Figure 3:**
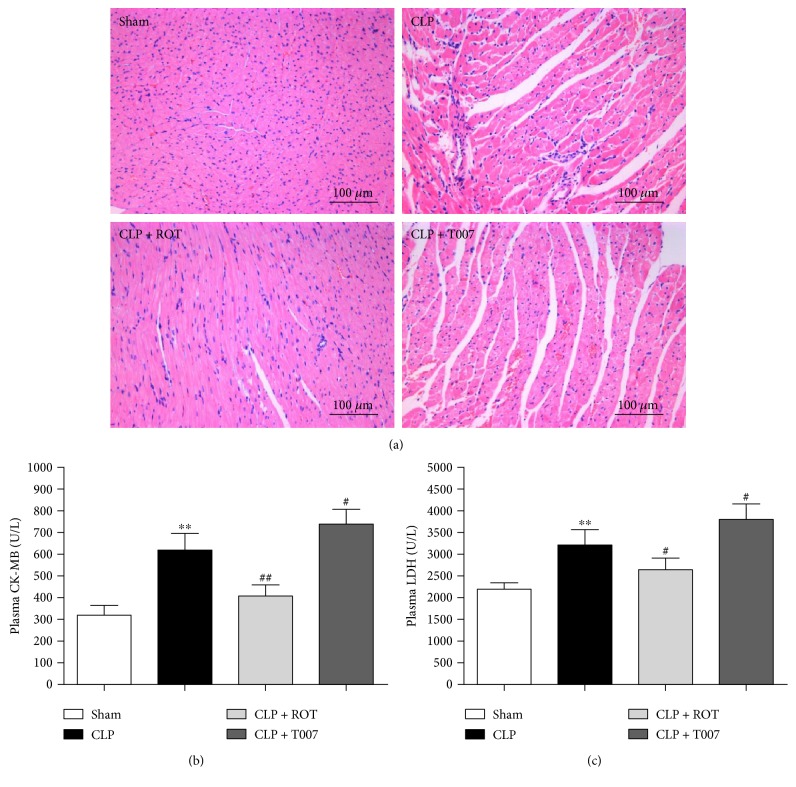
H&E staining of the myocardium and plasma levels of cardiotoxicity markers in septic rats. Myocardium and blood serum of rats subjected to CLP with or without pretreatment of rosiglitazone (ROT) or T007 were collected 18 hours after CLP. Myocardium architecture was visualized by (a) H&E staining. Cardiotoxicity was indicated by plasma levels of (b) cardiac injury marker, creatine kinase isoenzyme (CK-MB), and (c) necrosis marker, lactate dehydrogenase (LDH). ^∗∗^*P* < 0.01 versus sham. ^#^*P* < 0.05, ^##^*P* < 0.01 versus CLP. Data are presented as mean ± SEM. *n* = 6 per group.

Sepsis damages myocardium integrity which is associated with the increased plasma level of cardiotoxicity due to myocyte necrosis [27]. As demonstrated in the H&E staining of the myocardium (Figure 3(a)), normal architecture of the myocardium, shown in the sham group, was deformed after CLP. Pretreatment with rosiglitazone prevented the deformation, but T007 did not. In addition, myocardial injury and necrosis markers, namely, CK-MB and LDH, increased significantly after CLP (CLP versus sham, *P* < 0.01) (Figures 3(b) and 3(c)). Activation of PPAR-*γ* by rosiglitazone prevented the changes (CLP + ROT versus CLP, *P* < 0.01), while inhibition of PPAR-*γ* by T007 further enhanced them (CLP + T007 versus CLP, *P* < 0.05).

### 3.4. Activation of PPAR-*γ* Prevents Cardiomyocyte Apoptosis in Septic Rats

**Figure 4:**
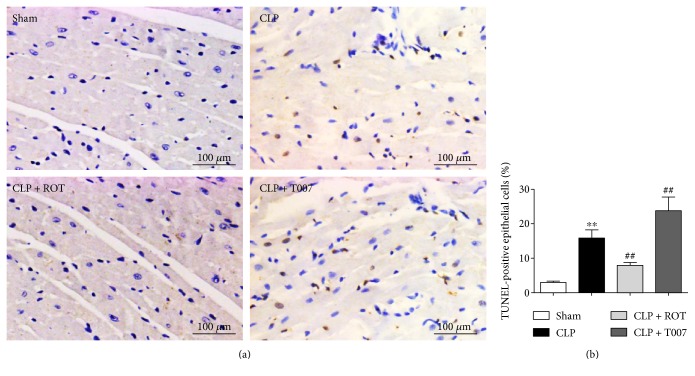
Cardiomyocyte apoptosis in septic rats. Myocardiums of rats subjected to CLP with or without pretreatment of rosiglitazone (ROT) or T007 were collected 18 hours after CLP. Apoptotic cardiomyocytes were assessed by terminal deoxynucleotidyl transferase dUTP nick end labeling (TUNEL). (a, b) TUNEL-positive cells were stained brown and counted. ^∗∗^*P* < 0.01 versus sham. ^##^*P* < 0.01 versus CLP. Data are presented as mean ± SEM. *n* = 6 per group.

Besides necrosis, apoptosis is also closely linked to sepsis-induced myocardial injury, which leads to heart failure [28]. As shown in Figure 4(), TUNEL-positive cells, stained brown, significantly increased in the myocardium of rats after CLP (CLP versus sham, *P* < 0.01). However, septic rats pretreated with rosiglitazone had significantly lower numbers of apoptotic cardiomyocytes than those without treatment (CLP + ROT versus CLP, *P* < 0.01). On the other hand, pretreatment with T007 further enhanced cardiomyocyte apoptosis in septic rats (CLP + T007 versus CLP, *P* < 0.01).

### 3.5. Enhanced Myocardial Expression and Binding Activity of PPAR-*γ* by Rosiglitazone Protect the Myocardium against Sepsis-Induced Cardiac Injury

**Figure 5:**
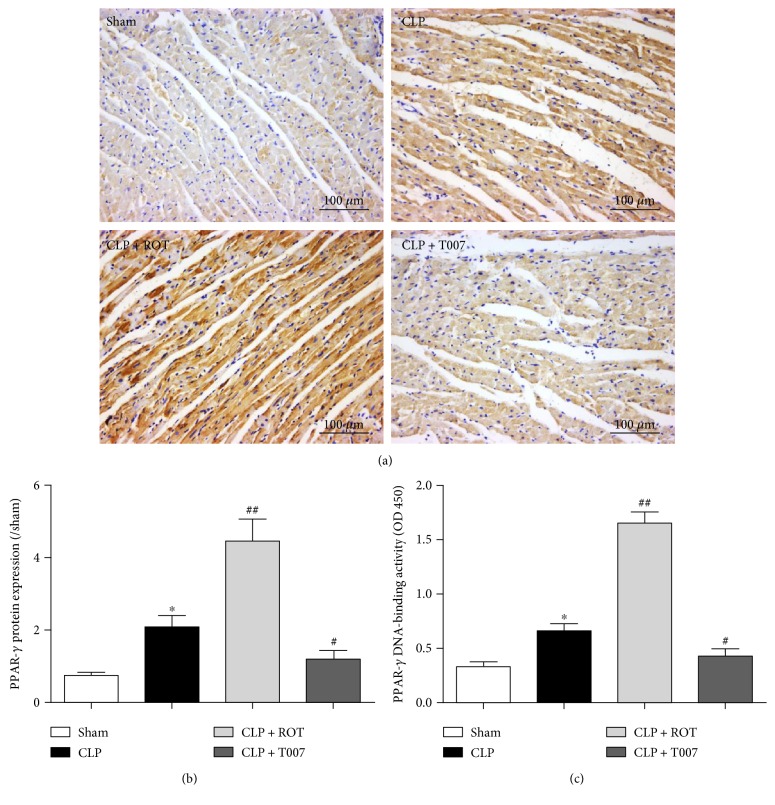
Protein expression and DNA binding activity of PPAR-*γ* in the myocardium of septic rats. PPAR-*γ* activity in the myocardium of rats subjected to CLP with or without pretreatment of rosiglitazone (ROT) and T007 was shown in terms of (a, b) protein expression stained with IHC assay and (c) DNA binding activity. ^∗^*P* < 0.01 versus sham. ^#^*P* < 0.05, ^##^*P* < 0.01 versus CLP. Data are presented as mean ± SEM. *n* = 6 per group.

As activation of PPAR-*γ* is proven beneficial in sepsis-induced myocardial injury, we then examined the protein expression and activity of PPAR-*γ* in the heart in sepsis. As shown in Figures 5(a) and 5(b), the IHC stain of the myocardium against PPAR-*γ* showed a destructed myocardium and doubled protein expression of PPAR-*γ* in septic rats compared to that in sham rats (CLP versus sham, *P* < 0.05). However, rosiglitazone-induced 5-fold increase of PPAR-*γ* compared to that in sham rats protected the integrity of myocardium from septic myocardial injury (CLP + ROT versus CLP, *P* < 0.01). On the other hand, inhibition of PPAR-*γ* by T007 significantly reduced PPAR-*γ* protein expression, which was associated with a damaged myocardium, in septic rats (CLP + T007 versus CLP, *P* < 0.05). DNA binding activity of PPAR-*γ* was similar to the expression profile of PPAR-*γ* after CLP and treatments with rosiglitazone or T007 (Figure 5(c)).

### 3.6. PPAR-*γ* Activation Alleviates Localized Myocardial Inflammation in Sepsis

**Figure 6:**
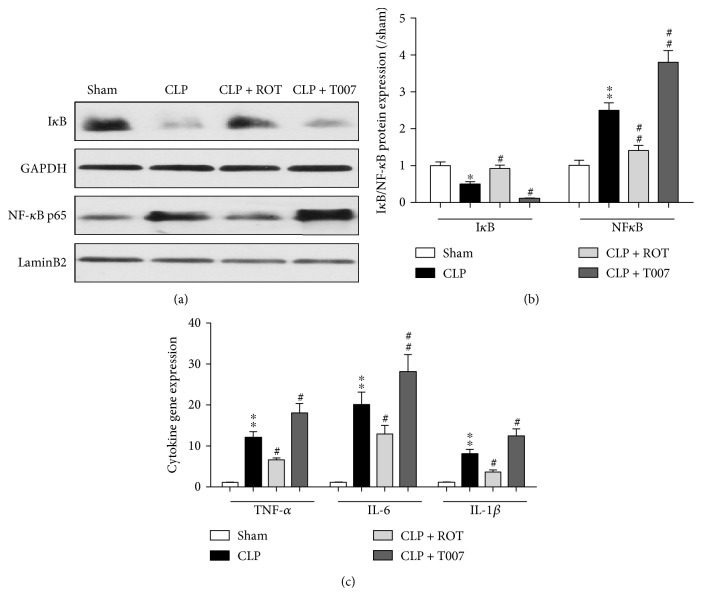
Expressions of inhibitor *κ*B (I*κ*B*α*), NF-*κ*B, and proinflammatory cytokines in the myocardium of septic rats. Rats were treated with or without rosiglitazone (ROT) or T007 before CLP and were sacrificed 18 hours after CLP. (a, b) Protein expressions of nuclear I*κ*B*α* and NF-*κ*B and (c) mRNA levels of proinflammatory cytokines, tumor necrosis factor-*α* (TNF-*α*), interleukin 6 (IL-6), and interleukin 1*β* (IL-1*β*) were assessed in the myocardium. ^∗^*P* < 0.01, ^∗∗^*P* < 0.01 versus sham. ^#^*P* < 0.05, ^##^*P* < 0.01 versus CLP. Data are presented as mean ± SEM. *n* = 6 per group.

Activation of NF-*κ*B following inhibitor *κ*B (I*κ*B*α*) protein degradation in response to proinflammatory cytokine stimulation plays a key role in prompting overt inflammation during sepsis [16, 29]. We found that septic rats had a marked decrease in I*κ*B*α* protein expression (CLP versus sham, *P* < 0.05) in the myocardium at 18 h after CLP (Figures 6(a) and 6(b)). The reduction of I*κ*B*α* was associated with a significant increase of NF-*κ*B p65 protein expression in the nucleus (CLP versus sham, *P* < 0.01) and upregulated gene expressions of proinflammatory cytokines TNF-*α*, IL-6, and IL-1*β* (CLP versus sham, *P* < 0.01) (Figure 6). Activation of PPAR-*γ* prior to CLP eradicated these changes while inhibition of PPAR-*γ* amplified them.

### 3.7. PPAR-*γ* Activation Inhibits Necroptosis in the Myocardium in Septic Rats

**Figure 7:**
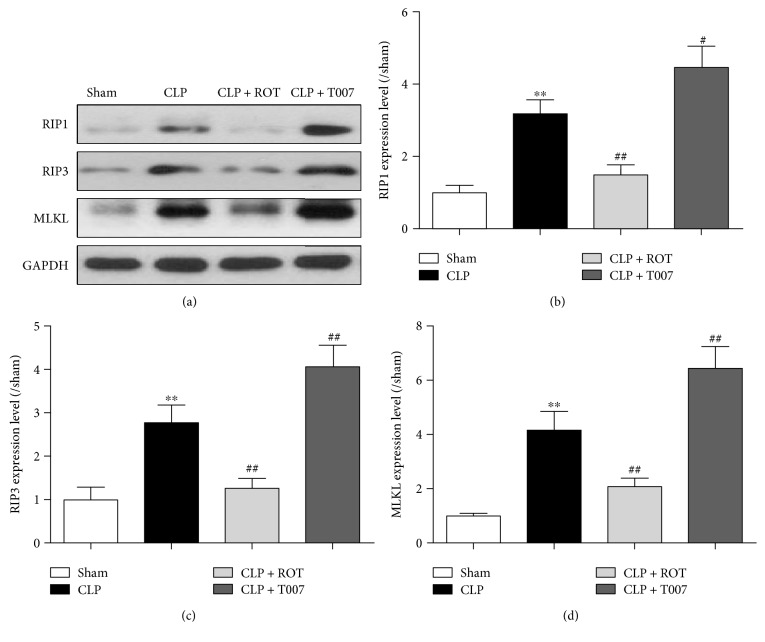
Necroptosis in the myocardium of rats after CLP. Myocardiums of rats subjected to CLP with or without pretreatment of rosiglitazone (ROT) or T007 were collected 18 hours after CLP. Necroptosis in myocardium was assessed by evaluating protein expressions of receptor-interacting protein kinase-1 (RIP1), RIP3, and mixed lineage kinase-like (MLKL) domain, which are the three key components of necroptosis. ^∗∗^*P* < 0.01 versus sham. ^#^*P* < 0.05, ^##^*P* < 0.01 versus CLP. Data are presented as mean ± SEM. *n* = 6 per group.

In order to assess whether PPAR-*γ* activation also inhibits inflammation-induced necroptosis, expressions of RIP1, RIP3, and MLKL, the three critical components of necroptosis [29], were evaluated in the myocardium. Here, we observed that PPAR-*γ* activation inhibits the CLP-induced necroptosis via downregulation of RIP1, RIP3, and MLKL. As shown in Figure 7, all three proteins were increased significantly in the myocardium of septic rats (CLP versus sham, *P* < 0.01). Pretreatment with rosiglitazone reversed these changes whereas T007 further augmented them.

## 4. Discussion

One of the major lethal consequences of sepsis is the development of cardiac dysfunction; as a result, a key predictor of survival in septic patients is their ability to recover from myocardial dysfunction [30]. In this study, we have found that activation of PPAR-*γ* before sepsis enhances expression and activity of PPAR-*γ* in myocardium and prevents septic myocardial dysfunction by reducing cardiac inflammation and suppressing multiple cell death pathways, namely, apoptosis, necroptosis, and necrosis. In particular, we have demonstrated that systemic administration of PPAR-*γ* is able to downregulate the localized expression of proinflammatory cytokines in the myocardium by stabilizing the I*κ*B*α*/NF-*κ*B complex in which NF-*κ*B is inhibited. Furthermore, PPAR-*γ* activation reduces myocardial expression of the RIP1/RIP3 necrosome complex and its downstream effector MLKL, suggesting that repression of necroptosis by PPAR-*γ* activation contributes to the improvement of myocardial function in sepsis. Importantly, these results suggest a pleiotropic role of PPAR-*γ* that it inhibits I*κ*B*α* degradation and necrosome formation via suppression of RIP1, which prevents unrestrained production of proinflammatory cytokines and cell death in myocardium, thus improving myocardial function during sepsis.

In the current study, CLP-induced sepsis caused lethal destruction to animal's cardiac function, which was associated with significantly altered hemodynamics (i.e., lowered MABP, dP/dt_max_, dP/dt_min_, LVSP, and increased LVEDP), hyperinflammatory response (i.e., upregulated TNF-*α*, IL-6, and IL-1*β*), and cardiotoxicity (i.e., increased CK-MB and LDH) due to cell death in the myocardium. Administration of PPAR-*γ* agonist, rosiglitazone, before CLP boosted the PPAR-*γ* level in the myocardium and prevented cardiac dysfunction, which was evidenced by protected cardiomyocyte integrity, enhanced cardiac function, reduced inflammation and cell death, and, finally, improved animal survival. On the other hand, we treated animals before CLP the PPAR-*γ* antagonist, T007, a potent and selective PPAR-*γ* antagonist which inhibits transcriptional activity of PPAR-*γ* by covalent modification of the PPAR-*γ* ligand-binding domain [31]. Pretreatment with T007 further exacerbated myocardial dysfunction, dropping the survival rate nearly to 0% by 72 hours after CLP, which suggests a key functional role of PPAR-*γ* in septic myocardial dysfunction by modulating the inflammatory and cell death pathways.

Although PPAR-*γ* has been documented as a negative regulator of inflammation [11, 12], whether this property plays a part in septic myocardial dysfunction is illusive. Transrepression of proinflammatory cytokines by PPAR-*γ* via inhibition of NF-*κ*B is a mechanism that has been closely linked to the PPAR-*γ*-mediated improvement of organ injury, including the heart, by eliminating systemic inflammation in sepsis [14, 15]. However, studies have shown that PPAR-*γ*-mediated prevention of cardiac dysfunction is not associated with attenuation of localized inflammation in the heart at the early stage of sepsis. Drosatos et al. reported that upon 7–9 hours after induction of sepsis by LPS, cardiac expression of TNF-*α* and IL-6 was not reduced by pretreatment of rosiglitazone or was not associated with cardiac PPAR-*γ*-mediated improvement of cardiac function [17, 18]. In our experiments, we found that at 18 hours after CLP, which is considered the acute phase of sepsis in this model [32], pretreatment of rosiglitazone prevented the degradation of NF-*κ*B inhibitor, I*κ*B*α*, hence repressing NF-*κ*B and subsequently downregulating the proinflammatory cytokines, namely, IL-1*β*, TNF-*α*, and IL-6, in the myocardium of septic rats. The discrepancy between Drosatos' [17] and our study may be due to the type of animal model and phase of sepsis, which implies that mechanism underlying PPAR-*γ*-mediated modulation of myocardial function varies by phase and the initiating agent of sepsis. Of note, LPS is not able to reproduce the hemodynamic changes observed in human sepsis while CLP shows that it closely mimics the cardiovascular state in human sepsis [33]. Therefore, our results may provide a relatively more clinically relevant indication that PPAR-*γ* activation prevents myocardial function by alleviation of sepsis-induced cardiac inflammation. In addition, the current result agrees with the previous study that, by reducing the degradation of I*κ*B*α*, PPAR-*γ* agonist repressed NF-*κ*B in the lung and reduced lung injury at 18 hours after CLP [16], suggesting that this protective mechanism mediated by PPAR-*γ* may be applicable to other organ injuries in sepsis.

Furthermore, we demonstrated that enhanced PPAR-*γ* activity in the myocardium primed the heart against septic cardiac dysfunction. The innate increase of protein expression and DNA binding of PPAR-*γ*, observed after CLP, was not able to compensate for the overwhelming inflammatory response, while the rosiglitazone-enhanced PPAR-*γ* expression (3-fold more than that of innate increase) ameliorated cardiac inflammation and dysfunction. This is in line with the previous study that PPAR-*γ* was upregulated shortly in the peritoneal or lung by 6 or 18 hours, respectively, after CLP but only prolonged enhancement of PPAR-*γ* by its agonists reduced inflammation and improved survival of septic animals [16, 34]. Although we did not catch a decrease of PPAR-*γ* in the myocardium by 18 hours after CLP, a previous study has reported reduced PPAR-*γ* protein expression in the lung by 20 hours after CLP [11]. Clinical observation also showed that in patients with sepsis, PPAR expression was downregulated and the magnitude of decrement correlated with the severity of the disease [35]. We suspect that the innate increase of PPAR-*γ* after CLP is compensatory in response to inflammation during early phase of sepsis and would be suppressed later as sepsis progresses. In addition, we showed that preinhibition of PPAR-*γ* significantly reduced its expression in the myocardium and further worsened myocardial dysfunction induced by CLP. Taken together, current results suggest that prolonged enhancement of PPAR-*γ* in myocardium represses cardiac inflammation and subsequently improves cardiac function in sepsis.

Besides unbridled inflammatory response, cell death as a result of hyperinflammation is a key pathological process in sepsis that contributes to the profound morbidity and mortality in this disorder [27, 28]. Strategies used to block apoptosis, necroptosis, or necrosis have been found beneficial in improving survival in animal models of sepsis [36]. However, while myocardial apoptosis and necrosis have been revealed as mechanisms of septic myocardial dysfunction, little is known about necroptosis, the regulated form of necrosis, in the pathogenesis of this lethal cardiac disease. Here, we showed that besides apoptosis and necrosis, necroptosis is also indicated in the pathogenesis of myocardial dysfunction, evidenced by increased number of TUNEL-positive cells and expression of RIP1/3 and MLKL in the myocardium and the elevated plasma level of LDH after CLP. Moreover, activation of PPAR-*γ* prevented these changes and improved cardiac function, while inhibition of PPAR-*γ* devastated the condition, suggesting a crucial role of PPAR-*γ* in the regulation of multiple cell death pathways initiated in the heart in sepsis. Of note, the repression of RIP1 by PPAR-*γ* agonist is particularly important in this process as RIP1 not only forms heterodimer with RIP3 to recruit MLKL for execution of necroptosis but also mediates the degradation of I*κ*B*α*, resulting in activation of NF-*κ*B and subsequent upregulation of cytokines, IL-1*β*, TNF-*α*, and IL-6 [37]. These cytokines promote infiltration of neutrophils and lead to tissue necrosis, favoring an overwhelming inflammatory response [38]. Hence, this repression of RIP1 by PPAR-*γ* further explains the reduced NF-*κ*B and associated inflammation in septic rats pretreated with rosiglitazone. During sepsis, inhibition of one dominant cell death pathway, namely, apoptosis, has been suggested insufficient because multiple cell death programs and crosstalk among them also occur in dying cells [39]. Therefore, the pleiotropic effect of PPAR-*γ* observed in this study, specifically the suppression of sepsis-induced multiple cell death programs in myocardium, may provide a potential insight for future research on the therapeutic value of PPAR-*γ* agonist.

The certain experiment limitation was recognized in the current study. For translational purpose, the present study used intraperitoneal injection as the administration route of PPAR-*γ* agonist and antagonist; however, this administration is systemic and may cause side effects that could contribute to the current results. Future cell culture study using PPAR-*γ* gene knockdown to confirm the role of PPAR-*γ* in apoptosis and necroptosis in myocardial cells is worth an investigation.

In summary, we have shown that pretreatment with rosiglitazone inhibits CLP-induced cardiac inflammation as well as apoptosis, necroptosis, and necrosis in the myocardium, resulting in the prevention of myocardial dysfunction and thus increased the survival in experimental polymicrobial sepsis. In this process, by reducing I*κ*B*α* degradation, PPAR-*γ* agonist inhibits the transcriptional activity of NF-*κ*B and subsequent overproduction of inflammatory cytokines, which are IL-1*β*, TNF-*α*, and IL-6. In particular, by reducing the expressions of RIP1/RIP3 necrosome and MLKL, PPAR-*γ* agonist suppresses necroptosis in the myocardium. These findings provide evidence that activation of PPAR-*γ* may be an effective intervention facilitating the prevention or recovery of septic myocardial dysfunction and therefore a promising therapeutic strategy against sepsis.
